# Socioeconomic and demographic factors are associated with dietary patterns in a cohort of young Brazilian adults

**DOI:** 10.1186/1471-2458-14-654

**Published:** 2014-06-26

**Authors:** Soraia Pinheiro Machado Arruda, Antônio Augusto Moura da Silva, Gilberto Kac, Marcelo Zubaran Goldani, Heloisa Bettiol, Marco Antônio Barbieri

**Affiliations:** 1 Department of Nutritional Sciences, State University of Ceará, Coordination of Nutrition Course, Av. Paranjana, 1700, Itapery, Fortaleza, CE 60.740-000, Brazil; 2 Department of Public Health, Federal University of Maranhão, Rua Barão de Itapary, 155, Centro, São Luís, Maranhão 65.020-070, Brazil; 3 Department of Applied Social Nutrition, Federal University of Rio de Janeiro, Av. Brigadeiro Trompowsky, s/n, bloco J, 2° andar, sala 29, Cidade Universitária, Rio de Janeiro 21.941-590, Brazil; 4 Department of Pediatrics and Puericulture, Faculty of Medicine, Federal University of Rio Grande do Sul, Rua Ramiro Barcellos, 2350, Bonfim, Porto Alegre, Rio Grande do Sul 90.035-003, Brazil; 5 Department of Puericulture and Pediatrics, Faculty of Medicine de Ribeirão Preto, University of São Paulo, Av. Bandeirantes, 3900, Monte Alegre, Ribeirão Preto, São Paulo 14.049-900, Brazil

**Keywords:** Dietary patterns, Socioeconomic factors, Young adults, Social mobility, Cohort study

## Abstract

**Background:**

The aim of the present study was to identify the main dietary patterns among young adults and to investigate the association of socioeconomic and demographic factors, and social mobility with dietary patterns.

**Methods:**

Data from the fourth follow-up of the 1978/79 Ribeirão Preto birth cohort study, Brazil, were used. A total of 2,061 young adults, whose mothers gave sociodemographic information at birth in 1978–79, provided sociodemographic and dietary data through a validated food frequency questionnaire in 2002–2004, when they were aged 23–25 years. Those whose caloric intake was outside of the ±3 standard deviation range were excluded, leaving 2,034 individuals. The dietary patterns were identified by principal component analysis followed by varimax orthogonal rotation. Poisson regression with robust estimation of variance was used to derive prevalence ratios (PR).

**Results:**

Four dietary patterns were identified: *healthy, traditional Brazilian, energy-dense* and *bar*. In the adjusted analysis, individuals with higher schooling (≥12 years) in adult life (PR = 1.51, 95% CI: 1.07-2.14) showed greater adherence whilst men (PR = 0.79, 95% CI: 0.68-0.93) had lower adherence to the *healthy* pattern. The highest adherence to the *traditional Brazilian* pattern was found for men (PR = 2.39, 95% CI: 2.04-2.80), mullatos (PR = 1.41, 95% CI: 1.21-1.64), households with ≥2 members, and for those with children (PR = 1.28, 95% CI: 1.07-1.55) while individuals with higher schooling in adulthood (≥12 years) (PR = 0.47, 95% CI: 0.34-0.65), higher family income in adulthood (≥20 MW) (PR = 0.57, 95% CI: 0.33-0.99) and higher family income at birth (≥6.1 MW) showed lower adherence. The *bar* pattern was positively associated with male sex (PR = 2.96, 95% CI: 2.47-3.55) and low schooling (≤8 years). The *energy-dense* pattern was not associated with any of the variables investigated. Social mobility was associated with the traditional Brazilian pattern. Men and women who were not poor at birth and remained so in adulthood showed lower adherence to this pattern (PR = 0.70, 95% CI: 0.53-0.94 for men and PR = 0.40, 95% CI: 0.20-0.80 for women).

**Conclusions:**

Four different dietary patterns were identified among young adults. Socioeconomic and demographic factors, and social mobility were associated with food choices.

## Background

Extensive epidemiological studies have been conducted in order to identify factors that might attenuate the burden of non-communicable diseases. Diet is one of the main modifiable risk factors [1,2].

However, the approach traditionally used by most studies within the field of nutritional epidemiology is based on the investigation of the specific effect of individual nutrients or foods on health. The variety of foods in a diet results in a complex combination of chemical compounds that may be antagonistic and that may compete with each other or may alter the bioavailability of other chemical compounds or nutrients. Thus, examining the individual effect of foods and nutrients on the risk of disease has serious limitations [3-6].

The analysis of food consumption based on dietary patterns helps disease prevention and treatment, and thus represents a fundamental tool for programs of nutritional intervention [7,8]. Some statistical techniques are employed for the identification of dietary patterns, in an attempt to examine the effect of overall diet on disease risk. The number of dietary variables is reduced and a combination of nutrients and foods that are easy to analyze is then used [4,9,10].

Although dietary patterns are not stable among different populations, many studies have identified similar patterns. The “western” pattern, that usually consists of red meat, whole dairy products, processed foods and refined grains, and the “prudent or healthy” pattern of fruits, vegetables, whole grains and fish, are among those commonly identified [9].

Demographic, socioeconomic, biological and nutritional factors have been associated with dietary patterns [5,11]. For example, some studies have shown that women follow healthier dietary patterns than men [9,12]. Similarly, the association of healthy dietary patterns with high income and educational level has been well established [11]. However, it is still unclear to what extent this association is related to current or early socioeconomic characteristics, especially during childhood, when dietary preferences are partially established [13].

Most of the studies investigating the dietary patterns of populations have been conducted in developed countries. It was only in the last decade that studies started to be conducted in developing countries such as Brazil [3,5,6,11,14-18]. Although most of the Brazilian studies have investigated adult populations, specific studies on young adults are scarce [16,18]. It is not known whether the same dietary patterns would be found in different regions of middle-income countries and if the associations of socioeconomic, demographic conditions and social mobility with these dietary patterns would be similar.

The objective of the present study was to identify the dietary patterns of young Brazilian adults and to investigate the association of socioeconomic and demographic factors, and social mobility with these patterns in a birth cohort from Ribeirão Preto, São Paulo, Brazil. This knowledge can guide planning and implementation of strategies to promote healthy eating practices aimed at preventing non-communicable diseases in later life.

## Methods

The study was based on the Ribeirão Preto, birth cohort study, started in 1978/79, involving children born in hospitals to mothers residing in the municipality. The current study used data collected in the first and fourth phase of the investigation, the latter covering the years from 2002 to 2004 [19]. Ribeirão Preto is the eighth most populous municipality in the State of São Paulo, in the Southeast region of Brazil. The population was 318,496 inhabitants in 1979, with 96.8% of them residing in the urban region. In 2003, the population was 534,828 inhabitants.

### Study population

In the study period, there were 9,396 live births in the maternity hospitals, corresponding to 98% of all liveborns in Ribeirão Preto, the rest being home births. Of those that were born in the maternity hospitals, we were not able to contact 235 (2.5) due to early discharge. Of the invited, 94 (1.0%) refused to participate in the study, leaving 9,067 individuals for inclusion. Then we excluded children whose families did not reside in the municipality (2,094) and twins (146), being the baseline sample at birth composed of 6,827 singletons.

In 2002/04 in the 4^th^ follow-up, when the study members were aged 23–25 years, 343 subjects have died before completing 20 years of age leaving 6,484 individuals. Attempts were then made to invite for medical examination a non-random sample based on one-in-three of this group. The first of every three names was selected from a list sorted by birth date in each geographic region and if unavailable, the next name down was selected. Based on the records of the Unified Health System and of private health care plans and on the contacts made in the 2nd and 3rd phase of the study, we were not able to locate 819, leaving 5,665 subjects. The losses due to refusal to participate in the study (209 cases), to death after 20 years of age (34 cases), imprisonment (31 cases) or failure to attend the interview (431 cases) corresponded to a total of 705 individuals. Losses were replaced using the same sampling frame, resulting in 2,063 young adults aged 23 to 25 years, corresponding to 31.8% of the 6,484 eligible subjects, participating in the 4th phase of the study of the Ribeirão Preto cohort [19]. Two subjects were excluded because they did not fill out the food frequency questionnaire and 27 because they reported energy intake outside the range ±3SD, leaving 2,034 cases for analysis.

The study was approved by the Research Ethics Committee of the Faculty of Medicine of Ribeirão Preto, University of São Paulo, and all subjects gave written informed consent to participate.

### Explanatory variables

At the time of birth, the mothers responded to a standardized, pre-coded and pre-tested questionnaire applied by trained interviewers [20]. The data considered for analysis in the present study were family income and maternal schooling. In young adulthood, the participants answered a questionnaire containing socioeconomic and demographic data. The variables considered were: sex, skin color, marital status, presence of children in the household, number of people in the household, family income in adult life, and schooling. Schooling was recorded as completed years of study. Monthly family income consisted of the sum of the income of family members residing in the household, converted to multiples of the Brazilian minimum wages (MW) for each period (corresponding to US$ 84.0 in 1978 and US$ 89.8 in 2003). Per capita family income was also calculated as the total family income divided by the number of family members. The information about family income obtained at the two time points was used to create the variable *social mobility*, which expresses life-long changes in family income. The family income of the first and fourth phase of the study was categorized into tertiles, with the first tertile being labeled *poor*, and the second and third tertiles *not poor*. Thus, social mobility was classified into four categories according to income tertiles at birth and adulthood: poor – poor, poor – not poor (ascending mobility), not poor – poor (descending mobility), and not poor – not poor.

### Dietary assessment

Dietary data were obtained by applying a Food Frequency Questionnaire (FFQ) validated for the Japanese-Brazilian community of São Paulo [21], with foods of Japanese origin being excluded. The reproducibility of the FFQ was assessed by repeated administration at one-month interval. The relative validity was evaluated by comparison with dietary intake obtained from four three-day weighed dietary records (DR), covering the same 12-month period assessed by the FFQ. The authors [21] conclude that this FFQ is reproducible and can be used to classify persons according to their nutrient intake over a one-year period.

The adapted FFQ contained 75 food items, the respective options of consumption frequency in the last year and the size of the mean reference portion so that the individual could estimate whether his regular consumed portion was small (smaller than the presented one), medium (equal to the presented one) or large (larger than the presented one). The questionnaire was applied, by a nutritionist, only to young adults at the 4th follow-up in 2002–2004, and a photograph album was used to help the subject estimate the portions consumed. Details of study methods have been described by Molina et al. [22].

The dietary variable used in the analysis was the daily consumption of each food in grams or milliliters. For the identification of the dietary patterns the 75 items of the FFQ were aggregated into 48 groups considering the similarity of the nutritional composition and the dietary habits of the municipality under study (Table 1). The food items cited by at least 80% of the subjects (for example, rice, beans, banana, chicken, beef, eggs and non-diet sodas) were kept separate.

**Table 1:** Description of the 48 food groups used in the analysis of dietary patterns of the 1978/79 Ribeirão Preto birth cohort (4th phase)

**Food group**	**Item**
Whole dairy products	Whole milk, whole yogurt with or without fruit, yellow cheese, curd cheese, cream cheese
Low-fat dairy products	Skimmed milk/semi-skimmed milk, skimmed yogurt, white cheese
Whole grain bread	Whole wheat bread/whole rye bread
White bread	French bread, loaf, sliced loaf, bun, cheese bread, others
Cookies	Sweet cookies, salty cookies, toasts
Breakfast cereals	Breakfast cereals, oatmeal, granola
Margarine	Margarine, light margarine, butter
Mayonnaise	Mayonnaise
Rice	White rice
Fried Potato	French fries, chips, fried manioc, fried polenta (fried corn mush)
Non-fried potato	Potato, manioc, cassava, polenta (corn mush), sweet potato, corn
Pasta	Pastas
Salty snacks	Fried snacks, salty cake, salty pie, pizza
Flours	*Farofa* (fried manioc flour with butter or margarine and salt), corn flour
Orange	Orange, tangerine
Banana	Banana
Apple	Apple, pear
Papaya/melon	Papaya, melon, watermelon
Other fruits	Grape, pineapple, guava, avocado, mango, persimmon, other fruits
Fruit juice	Orange juice, other fruit juices
Bean	Bean
Other legumes	Peas, lentils, others
Leafy vegetables	Lettuce, endive, watercress, arugula, chicory, cabbage, chard, kale, spinach
Other vegetables	Beet, pod, chayote, zucchini, eggplant
Tomato	Tomato
Broccoli	Broccoli, cauliflower
Soup	Soups
Beef	Beef with or without fat
Pork meat	Pork meat with or without fat
Bacon	Bacon, lard, crackling
Chicken	Chicken meat, chester, turkey meat
Fish	Fish
Organ meat	Organ meats from cattle, poultry or pork
Sea food	Shrimp, prawn, other sea food
Cold cuts	Sausage, ham, mortadela, salami
Egg	Fried egg, boiled egg
Coffee	Coffee with or without sugar
Teas	Black tea, herbal tea
Beer	Beer
Liquors	*Cachaça* (Brazilian rum made with sugar cane), whisky, other liquors
Wine	Wine
Industrialized fruit juices	Industrialized fruit juices
Diet sodas	Diet sodas, light sodas
Soda	Soda, pop, coke
Honey	Honey, jelly
Desserts	Cakes, pies, parfaits, ice cream, milk shake, pudding, fruit sweets
Chocolate	Chocolates, b*rigadeiro* (Brazilian sweet made with condensed milk and chocolate)
Nuts/seeds	Cashew nut, peanut, nuts

### Statistical analysis

The method of principal component analysis (PCA) was used to identify the dietary patterns, followed by the varimax method of orthogonal rotation to simplify interpretation of extracted factors. Estimation was based on the correlation matrix. The appropriateness of the dataset for applying the PCA was confirmed by the Kaiser-Meyer-Olkin (KMO) measure of sampling adequacy and the Bartlett test of sphericity [23]. The number of retained factors was defined based on the following criteria: components with eigenvalues higher than 1.0 (indicating that the factor explains more of the total variance than each of the original variables separately), the Cattell scree plot and the conceptual meaning of the patterns identified [9]. Each principal component was interpreted based on foods with factor loadings (correlation coefficients between the dietary variables and the factors) ≥ 0.3 or ≤ −0.3, which are considered to make an important contribution to the pattern [8,12]. Negative loadings within a component indicate an inverse association of the food item and positive loadings indicate a direct association [15].

To determine the stability of the factors the set of observations was divided at random into two sub-sets and the same criterion was applied to each one of them. The factorial structures detected for the subgroups were compared to those of the entire sample. The principal components were labeled on the basis of the nutritional composition of foods of each factor. Each individual received a score for each retained factor, calculated by summing the standardized values of the items (food groups) weighted by their absolute scoring coefficients (factor loadings). The dietary patterns were categorized into quartiles. The upper quartile of the distribution represented the greatest adherence to the pattern.

Poisson regression with robust estimation of variance was used in bivariate and multivariable analysis to estimate the prevalence ratios (PR) of the independent variables (socioeconomic and demographic factors) in relation to the outcomes, with the dependent variables (dietary patterns) being classified as dichotomous: 1st, 2^nd^ and 3^rd^ quartiles of consumption and high consumption (4^th^ quartile). We used Poisson regression to estimate the PR instead of the odds ratio because when the outcome is not a rare event (>10%) by using odds ratio we tend to overestimate associations [24]. Variables whose p-values were <0.20 were included in the multivariable models. Interaction terms between sex and social mobility were tested and when significant models were presented stratified by sex. Since schooling is highly correlated with income, models with and without adjustment for schooling were fitted. Adjustment for schooling at two points in life may inadequately remove the possible effect of social mobility (measured by means of changes in income) on the dietary patterns. Estimates of 95% confidence intervals (CI) of the PR were also calculated. The level of significance was set at 0.05. The statistical analyses were performed using the Stata software, version 10.0. To test for consistency, analyses were also performed using per capita family income.

## Results

The study sample consisted of 52.2% women, 67.9% singles, and 66.3% whites. Only 15.4% had up to eight years of schooling, while among their mothers this percentage was 73.0% at the time of their birth. The young adults also reported a higher current family income than the family income at the time of their birth, with the value being lower than three MW only in 10.6% of cases. Despite this, however, only 18.9% of the participants presented ascending social mobility (Table 2).

**Table 2:** Socioeconomic and demographic characteristics of 23 to 25-year-old adults of the Ribeirão Preto birth cohort (4th phase: 2002–2004) according to sex

**Characteristics**	**Women (n = 1,062)**	**Men (n = 972)**		**Total (n = 2,034)**
	**n (%)**	**n (%)**	**p value**	**n (%)**
*Skin color*				
White	731 (68.8)	618 (63.6)	0.086	1,349 (66.3)
Black	45 (4.3)	43 (4.4)		88 (4.3)
Mullato	272 (25.6)	295 (30.4)		567 (27.9)
Yellow	14 (1.3)	16 (1.7)		30 (1.5)
*Schooling in adulthood (years)*				
0 – 8	156 (14.7)	158 (16.3)	0.173	314 (15.4)
9 – 11	521 (49.1)	499 (51.3)		1020 (50.2)
≥ 12	385 (36.2)	315 (32.4)		700 (34.4)
*Maternal schooling at birth (years)*^*a*^				
0 – 4	477 (45.8)	432 (45.4)	0.598	909 (45.6)
5 – 8	296 (28.4)	251 (26.4)		547 (27.4)
9 – 11	163 (15.6)	162 (17.0)		325 (16.3)
≥ 12	106 (10.2)	107 (11.2)		213 (10.7)
*Marital status*				
Married/Cohabiting	401 (37.8)	251 (25.8)	< 0.001	652 (32.1)
Single	661 (62.2)	721 (74.2)		1,382 (67.9)
*Family income in adulthood (MW)*				
< 3.0	125 (11.8)	90 (9.3)	< 0.001	215 (10.6)
3.0 – 4.9	251 (23.6)	207 (21.3)		458 (22.5)
5.0 – 9.9	340 (32.0)	283 (29.1)		623 (30.6)
10.0 – 19.9	177 (16.7)	219 (22.5)		396 (19.5)
≥ 20.0	79 (7.4)	113 (11.6)		192 (9.4)
Missing	90 (8.5)	61 (6.2)		150 (7.4)
*Family income at birth (MW)*				
≤ 1.0	168 (15.8)	137 (15.0)	0.198	305 (15.0)
1.1 – 3.0	307 (28.9)	289 (29.7)		596 (29.3)
3.1 – 6.0	200 (18.8)	207 (21.3)		407 (20.0)
6.1 – 10.0	86 (8.1)	86 (8.9)		172 (8.5)
> 10.0	92 (8.7)	96 (9.9)		188 (9.2)
Missing	209 (19.7)	157 (16.2)		366 (18.0)
*Number of people in the household*				
1	19 (1.8)	16 (1.7)	0.795	35 (1.7)
2 – 4	712 (67.0)	640 (65.9)		1,352 (66.5)
≥ 5	331 (31.2)	316 (32.5)		647 (31.8)
*Children in the household*				
Yes	708 (66.7)	774 (79.6)	< 0.001	1,482 (72,9)
No	354 (33.3)	198 (20.4)		552 (27,1)
*Social mobility*^*b*^				
Poor – poor	171 (21.9)	135 (17.6)	0.138	306 (19.8)
Poor – not poor (ascending mobility)	144 (18.5)	149 (19.4)		293 (18.9)
Not poor – poor (descending mobility)	111 (14.2)	103 (13.4)		214 (13.8)
Not poor – not poor	354 (45.4)	381 (49.6)		735 (47.5)

MW, minimum wages: monthly MW at birth in 1978 was equivalent to US$ 84.0, MW in 2003 was equivalent to US$ 89.8.

p value based on the chi-square test.

^*a*^Total n = 1,994, n of men = 952, n of women = 1,042.

^*b*^Total n = 1,548, n of men = 768, n of women = 780.

The KMO coefficient (0.7448) and the Bartlett test of sphericity (p <0.001) indicated satisfactory confidence for the execution of factor analysis. Sixteen factors with eigenvalues ≥1.0 were retained and the inflexion of the scree plot indicated three factors. Analysis of the conceptual meaning of these factors identified four principal dietary patterns, which explained 20.92% of the total variance. Only these four retained patterns presented eigenvalues ≥1.5 (Table 3). The factorial structures detected for the subgroups were similar to those of the entire sample, confirming the stability of the factors.

**Table 3:** Distribution of factor loadings of the principal dietary patterns identified among 23 to 25-year-old adults of the Ribeirão Preto birth cohort (4th phase: 2002–2004)

**Food groups**	**Dietary patterns**			
	***Healthy***	***Traditional Brazilian***	***Bar***	***Energy-dense***
Leafy vegetables	0.585			
Papaya, melon	0.522			
Apple, pear	0.495			
Yellow vegetables	0.484			
Broccoli	0.458			
Fish	0.446			
Tomato	0.421			
Potato, manioc and polenta, not fried	0.398			
Orange, tangerine	0.389			
Chicken	0.358			
Breakfast cereals	0.352			
Banana	0.335			
Fruit juice	0.318			
Peas, lentils	0.312			
Beans		0.695		
Rice		0.690		
Margarine		0.340		
Beef		0.301		
Low-fat dairy foods		- 0.397		
Whole grain breads		- 0.346		
Diet/light sodas		- 0.336		
Beer			0.592	
Salty snacks			0.519	
Distilled beverages (Brazilian rum - cachaça, whiskey, vodka)			0.479	
Porkmeat			0.402	
Sausages			0.351	
Eggs			0.343	
Bacon			0.342	
Seafood			0.335	
Mayonnaise			0.308	
Desserts, cakes, pies, ice cream, sweets				0.572
White breads, croissant, cheese bread				0.451
Sweet or salty cookies, toast				0.449
Chocolates				0.434
Popcorn, chips				0.386
Fried potato, manioc and polenta				0.345
Whole milk dairy products				0.303
*Explained variance (%)*	6.56	5.15	4.78	4.43
*Eigenvalue*	3.15	2.47	2.29	2.12

Only foods with factor loadings ≥ 0.3 or ≤ − 0.3 were shown.

Total explained variance = 20.92%.

The dietary patterns were defined as follows: *healthy* (vegetables, fruits, peas and other legumes, fish, non-fried potatoes, manioc and polenta, chicken and breakfast cereals); *traditional Brazilian* (beans, rice, margarine and beef and a negative factor loading for low-fat dairy foods, whole grain bread and diet sodas); *bar* (alcoholic beverages, salty snacks, pork meat, sausages, eggs, bacon, seafood and mayonnaise) and *energy-dense* (desserts, white bread, cookies, chocolates, popcorn*/*chips, fried potatoes, manioc and polenta and whole milk dairy products). The *healthy* and *traditional Brazilian* patterns explained the greatest proportion of total variance (6.56% and 5.15%, respectively) (Table 3).

After adjustment in multivariable analysis, women and individuals with higher schooling in adulthood (≥12 years) showed greater adherence to the healthy pattern. Men, mullatos, those who have children and living in households with ≥2 members showed the highest adherence to the traditional Brazilian pattern, whereas individuals with higher schooling in adulthood (≥12 years), higher family income in adulthood (≥20 MW) and higher family income at birth (≥6.1 MW) showed lower adherence. Men and individuals with lower schooling (≤8 years) showed greater adherence to the *bar* pattern. The *energy-dense* pattern was not associated with any of the variables investigated (Table 4).

**Table 4:** Non-adjusted and adjusted prevalence ratios (PR) and confidence intervals (95% CI) for the association of socioeconomic and demographic variables with the dietary patterns identified among 23 to 25-year-old adults of the Ribeirão Preto birth cohort (4th phase: 2002–2004)

**Variable**	***Healthy***		***Traditional Brazilian***		***Bar***		***Energy dense***	
	**Non-adjusted PR (95% CI)**	**Adjusted PR (95% CI)**	**Non-adjusted PR (95% CI)**	**Adjusted PR (95% CI)**	**Non-adjusted PR**	**Adjusted PR (95% CI)**	**Non-adjusted PR (95% CI)**	**Adjusted PR (95% CI)**
Sex	p = 0.011	p = 0.004	p < 0.001	p < 0.001	p < 0.001	p < 0.001	p = 0.625	—
Female	Reference	Reference	Reference	Reference	Reference	Reference	Reference	—
Male	0.82 (0.71 – 0.96)	0.79 (0.68 – 0.93)	2.99 (1.42 – 3.71)	2.39 (2.04 – 2.80)	4.33 (3.47 – 5.41)	2.96 (2.47 – 3.55)	0.95 (0.78 – 1.16)	—
*Skin color*	p = 0.009	p = 0.391	p < 0.001	p < 0.001	p = 0.645	—	p = 0.103	p = 0.103
White	Reference	Reference	Reference	Reference	Reference	—	Reference	Reference
Black	0.64 (0.37 – 1.10)	0.86 (0.56 – 1.33)	2.66 (1.69 – 4.19)	1.26 (0.95 – 1.67)	1.20 (0.74 – 1.93)	—	1.44 (0.91 – 2.89)	1.44 (0.91 – 2.89)
Mullato	0.70 (0.55 – 0.89)	0.93 (0.77 – 1.14)	2.83 (2.28 – 3.53)	1.41 (1.21 – 1.64)	1.01 (0.80 – 1.26)	—	0.89 (0.71 – 1.12)	0.89 (0.71 – 1.12)
Yellow	0.53 (0.20 – 1.40)	0.49 (0.20 – 1.22)	1.11 (0.45 – 2.74)	1.37 (0.66 – 2.86)	0.60 (0.23 – 1.59)	—	0.45 (0.15 – 1.30)	0.45 (0.15 – 1.30)
*Schooling in adulthood (years)*	p < 0.001	p = 0.068	p < 0.001	p < 0.001	p = 0.003	p < 0.001	p = 0.539	—
0 – 8	Reference	Reference	Reference	Reference	Reference	Reference	Reference	—
9 – 11	1.59 (1.13 – 2.23)	1.34 (0.99 – 1.80)	0.61 (0.47 – 0.79)	0.91 (0.77 – 1.07)	0.62 (0.47 – 0.83)	0.67 (0.55 – 0.82)	0.95 (0.71 – 1.27)	—
≥ 12	2.59 (1.84 – 3.66)	1.51 (1.07 – 2.14)	0.13 (0.09 – 0.18)	0.47 (0.34 – 0.65)	0.79 (0.59 – 1.06)	0.72 (0.56 – 0.93)	0.86 (0.63 – 1.17)	—
*Maternal schooling at birth (years)*	p < 0.001	p = 0.271	p < 0.001	p = 0.160	p = 0.041	p = 0.165	p = 0.379	—
0 – 4	Reference	Reference	Reference	Reference	Reference	Reference	Reference	—
5 – 8	1.35 (1.05 – 1.73)	1.17 (0.95 – 1.42)	0.55 (0.43 – 0.70)	0.84 (0.71 – 1.01)	1.27 (0.99 – 1.63)	1.23 (1.02 – 1.48)	1.10 (0.86 – 1.40)	—
9 – 11	1.61 (1.20 – 2.14)	1.18 (0.93 – 1.49)	0.35 (0.25 – 0.48)	0.85 (0.65 – 1.12)	1.24 (0.93 – 1.66)	1.09 (0.86 – 1.37)	0.91 (0.68 – 1.23)	—
≥ 12	2.20 (1.59 – 3.04)	1.30 (0.97 – 1.73)	0.14 (0.08 – 0.25)	0.72 (0.43 – 1.19)	1.54 (1.10 – 2.14)	1.08 (0.80 – 1.44)	0.81 (0.56 – 1.16)	—
*Marital status*	p = 0.002	p = 0.475	p = 0.002	p = 0.218	p < 0.001	p = 0.140	p = 0.595	—
Single	Reference	Reference	Reference	Reference	Reference	Reference	Reference	—
Married/Cohabiting	0.71 (0.56 – 0.88)	0.93 (0.75 – 1.14)	1.39 (1.13 – 1.72)	0.89 (0.74 – 1.07)	0.66 (0.53 – 0.83)	0.87 (0.72 – 1.05)	0.94 (0.76 – 1.17)	—
*Family income in adulthood (MW)*	p < 0.001	p = 0.046	p < 0.001	p = 0.090	p < 0.001	p = 0.195	p = 0.372	—
< 3.0	Reference	Reference	Reference	Reference	Reference	Reference	Reference	—
3.0 – 4.9	0.77 (0.52 – 1.15)	0.74 (0.54 – 1.02)	0.82 (0.59 – 1.15)	0.93 (0.74 – 1.06)	0.90 (0.61 – 1.33)	0.92 (0.69 – 1.23)	0.83 (0.58 – 1.19)	—
5.0 – 9.9	1.07 (0.74 – 1.55)	0.85 (0.63 – 1.15)	0.52 (0.38 – 0.73)	0.93 (0.75 – 1.15)	1.10 (0.76 – 1.59)	1.06 (0.80 – 1.40)	0.80 (0.57 – 1.13)	—
10.0 – 19.9	1.54 (1.05 – 2.27)	1.02 (0.73 – 1.42)	0.29 (0.20 – 0.43)	0.86 (0.69 – 1.09)	1.10 (0.74 – 1.63)	0.94 (0.68 – 1.29)	0.74 (0.51 – 1.08)	—
≥ 20	2.03 (1.32 – 3.13)	1.08 (0.74 – 1.56)	0.13 (0.07 – 0.23)	0.57 (0.33 – 0.99)	2.07 (1.35 – 3.20)	1.27 (0.90 – 1.81)	0.61 (0.39 – 0.97)	—
Missing	0.78 (0.46 – 1.31)	0.68 (0.44 – 1.03)	0.69 (0.45 – 1.08)	0.99 (0.74 – 1.32)	1.23 (0.76 – 1.99)	1.12 (0.79 – 1.59)	0.91 (0.57 – 1.44)	—
*Family income at birth (MW)*	p = 0.004	p = 0.787	p < 0.001	p = 0.006	p = 0.020	p = 0.207	p = 0.219	—
≤ 1.0	Reference	Reference	Reference	Reference	Reference	Reference	Reference	—
1.1 – 3.0	1.09 (0.78 – 1.52)	0.93 (0.71 – 1.22)	0.78 (0.59 – 1.04)	0.99 (0.83 – 1.19)	1.04 (0.75 – 1.45)	1.00 (0.78 – 1.28)	1.07 (0.79 – 1.47)	—
3.1 – 6.0	1.37 (0.98 – 1.94)	0.95 (0.71 – 1.28)	0.44 (0.32 – 0.62)	0.83 (0.65 – 1.05)	1.03 (0.72 – 1.47)	0.93 (0.71 – 1.2)	1.02 (0.73 – 1.44)	—
6.1 – 10.0	1.35 (0.87 – 2.08)	0.80 (0.56 – 1.15)	0.18 (0.10 – 0.31)	0.57 (0.35 – 0.94)	1.51 (0.99 – 2.30)	1.23 (0.88 – 1.71)	1.00 (0.65 –1.53)	—
> 10.0	2.04 (1.36 – 3.07)	0.98 (0.69 – 1.40)	0.09 (0.05 – 0.18)	0.37 (0.19 – 0.69)	1.76 (1.17 – 2.64)	1.27 (0.91 – 1.75)	0.64 (0.41 –1.01)	—
Missing	1.10 (0.76 – 1.59)	0.89 (0.66 – 1.20)	0.49 (0.35 – 0.69)	0.82 (0.65 – 1.03)	1.22 (0.85 – 1.75)	1.16 (0.87 – 1.51)	0.88 (0.62 – 1.25)	—
*Number of people in the household*	p = 0.744	—	p = 0.003	p = 0.122	p = 0.137	p = 0.271	p = 0.774	—
1	Reference	—	Reference	Reference	Reference	Reference	Reference	—
2 – 4	1.26 (0.65 – 2.47)	—	8.54 (1.23 – 59.12)	6.97 (1.02 – 47.57)	0.65 (0.42 – 1.01)	0.73 (0.48 – 1.11)	1.25 (0.64 – 2.43)	—
≥ 5	1.23 (0.62 – 2.42)	—	9.57 (1.38 – 66.38)	7.21 (1.06 – 49.18)	0.71 (0.45 – 1.11)	0.78 (0.51 – 1.20)	1.27 (0.64 – 2.49)	—
*Children in the household*	p = 0.001	p = 0.435	p < 0.001	p = 0.007	p = 0.921	—	p = 0.365	—
No	Reference	Reference	Reference	Reference	Reference	—	Reference	—
Yes	0.73 (0.61 – 0.88)	0.91 (0.72 – 1.15)	1.50 (1.29 – 1.75)	1.28 (1.07 – 1.55)	0.99 (0.84 – 1.17)	—	0.92 (0.78 – 1.10)	—

MW, minimum wages: monthly MW at birth was equivalent to US$ 84.0 MW in 1978 and to US$ 89.8 in 2003.

p value for trend referring to the maximum likelihood ratio test obtained by Poisson regression.

^**a**^Adjusted for sex, skin color, maternal schooling at birth, schooling in adulthood, marital status, family income at birth, family income in adulthood, and presence of children in the household.

^**b**^Adjusted for sex, skin color, maternal schooling at birth, schooling in adulthood, marital status, family income at birth, family income in adulthood, number of people in the household and presence of children in the household.

^**c**^Adjusted for sex, maternal schooling at birth, schooling in adulthood, marital status, family income at birth, family income in adulthood and number of people in the household.

^**d**^Adjusted for skin color.

Social mobility was not associated with the *healthy, traditional Brazilian* and *bar* patterns (Table 5). The results for social mobility in the models without adjustment for schooling were similar to those found for the analysis with adjustment for schooling. A significant interaction between sex and social mobility (p = 0.009) was detected only for the *traditional Brazilian* dietary pattern. Among women and men, those who were not poor at birth and remained so in adulthood, the category ‘not poor - not poor’, showed lower adherence to this pattern, with or without adjustment for schooling (Table 6).

**Table 5:** **Non-adjusted and adjusted prevalence ratios (PR) and confidence intervals (95% CI) for the association of social mobility with the *healthy, bar *** **and energy dense dietary patterns identified in 23 to 25-year-old adults of the Ribeirão Preto birth cohort (4th phase: 2002–2004)**

**Social mobility**	**Healthy pattern**		**Bar pattern**		**Energy dense pattern**	
	**Non-adjusted PR (95% CI)**	**Adjusted PR (95% CI)**^**a**^	**Non-adjusted PR (95% CI)**	**Adjusted PR (95% CI)**^**b**^	**Non-adjusted PR (95% CI)**	**Adjusted PR (95% CI)**^**c**^
	**p = 0.001**	**p = 0.541**	**p = 0.185**	**p = 0.622**	**p = 0.331**	**p = 0.664**
Poor – poor	Reference	Reference	Reference	Reference	Reference	Reference
Poor – not poor	1.08 (0.73 – 1.58)	0.93 (0.68 – 1.26)	1.18 (0.81 – 1.73)	1.09 (0.82 – 1.46)	1.14 (0.78 – 1.66)	1.11 (0.83 – 1.48)
Not poor – poor	0.91 (0.59 – 1.41)	0.82 (0.58 – 1.17)	0.95 (0.62 – 1.45)	0.88 (0.64 – 1.22)	0.92 (0.61 – 1.41)	0.97 (0.70 – 1.33)
Not poor – not poor	1.62 (1.19 – 2.22)	1.01 (0.76 – 1.36)	1.31 (0.96 – 1.80)	0.99 (0.75 – 1.33)	1.23 (0.90 – 1.69)	1.13 (0.86 – 1.49)

Total n = 1,549, n of men = 769, n of women = 780.

p value for trend referring to the maximum likelihood ratio test obtained by Poisson regression.

^**a**^Adjusted for sex, skin color, maternal schooling at birth, schooling in adulthood, marital status and presence of children in the household.

^**b**^Adjusted for sex, maternal schooling at birth, schooling in adulthood, marital status and number of people in the household.

^**c**^Adjusted for skin color.

**Table 6:** Non-adjusted and adjusted prevalence ratios (PR) and confidence intervals (95% CI) for the association of social mobility with the traditional Brazilian dietary pattern stratified by sex, among 23 to 25-year-old adults of the Ribeirão Preto birth cohort (4th phase: 2002–2004)

**Social mobility**	**Men**			**Women**		
	**Non-adjusted PR (95% CI)**	**Adjusted PR (95% CI)**^**a**^	**Adjusted PR (95% CI)**^**b**^	**Non-adjusted PR (95% CI)**	**Adjusted PR (95% CI)**^**a**^	**Adjusted PR (95% CI)**^**b**^
	**p < 0.001**	**p = 033**	**p < 0.001**	**p < 0.001**	**p = 0.082**	**p < 0.001**
Poor – poor	Reference	Reference	Reference	Reference	Reference	Reference
Poor – not poor	0.59 (0.37 – 0.94)	0.90 (0.69 – 1.16)	0.83 (0.64 – 1.07)	0.62 (0.36 – 1.05)	0.86 (0.56 – 1.32)	0.83 (0.54 – 1.28)
Not poor – poor	0.78 (0.47 – 1.30)	1.07 (0.82 – 1.40)	1.01 (0.77 – 1.31)	0.56 (0.31 – 1.01)	0.79 (0.50 – 1.27)	0.72 (0.45 – 1.13)
Not poor – not poor	0.24 (0.16 – 0.36)	0.70 (0.53 – 0.94)	0.50 (0.38 – 0.66)	0.11 (0.06 – 0.21)	0.40 (0.20 – 0.80)	0.23 (0.12 – 0.44)

Total n = 1,549, n of men = 769, n of women = 780.

p value for trend referring to the maximum likelihood ratio test obtained by Poisson regression.

^*a*^Adjusted for skin color, number of people in the household, presence of children in the household, maternal schooling at birth, schooling in adulthood and marital status.

^*b*^Adjusted for skin color, number of people in the household, presence of children in the household and marital status.

In analysis using per capita family income results did not change appreciably.

## Discussion

Four principal dietary patterns were identified among the 2,034 young adults of the Ribeirão Preto birth cohort, denoted *healthy, traditional Brazilian, bar* and *energy-dense*. The variance explained by these factors (20.92%) was lower than that reported in other studies which also retained four components [17,25]. The decision to include a large number of food items in the principal component analysis may have contributed to the lower percentage of explanation of total variance [9,12]. In the adjusted analysis, women and individuals with higher schooling (≥12 years) in adult life showed greater adherence to the *healthy* pattern. The highest adherence to the *traditional Brazilian* pattern was detected for men, mullatos, households with ≥2 members, and for those who have children, while individuals with higher schooling in adulthood (≥12 years), higher family income in adulthood (≥20 MW) and higher family income at birth (≥6.1 MW) showed lower adherence. The *bar* pattern was positively associated with male sex and low schooling (≤8 years). The *energy-dense* pattern was not associated with any of the variables investigated. Social mobility was associated with the *traditional Brazilian pattern. Men and women* who were not poor at birth and remained so in adulthood showed lower adherence to this pattern.

The dietary pattern denoted *healthy* explained the greatest percentage of variance. Its composition is similar to that of other patterns reported in the literature with the same denomination [5,10,26-28] or even with others such as ‘prudent’ [15,25,29], ‘nutrient dense’ [30] and ‘health aware’ [31]. This pattern is characterized by the presence of foods rich in vitamins, minerals, fibers, and unsaturated fats and with low contents of sugars, trans and saturated fats [25]. The *traditional Brazilian* pattern involved traditional foods of the Brazilian diet, as it was also observed in other studies [3,6,14,15]. In a similar study conducted on young adults (23 years) from Pelotas, Rio Grande do Sul, Brazil, Olinto et al. [16] defined a similar pattern as ‘*common Brazilian*’, and Gimeno et al. [17], in a study of adults aged 30 years or older from Ribeirão Preto, called it ‘popular’. The ‘traditional’ denomination is habitually used in studies from other countries, but the foods that compose this diet vary according to the culture of each country [9].

The dietary pattern denoted *bar* consisted of alcoholic beverages and foods commonly served in bars. No pattern with this denomination was found in the literature. However, some studies have identified factors containing alcoholic beverages in their composition [9,16,26,31,32]. The fourth pattern identified was called *energy-dense* because it is rich in sugars and saturated and trans fats. Patterns with similar compositions have been designated in various ways in other studies: ‘obesogenic’ [17], ‘energy dense’ [30], ‘snack’ [15] and ‘dairy foods and desserts’ [6].

It should be pointed out that many studies have identified a pattern defined, in most cases, as western [25,26,33], which represents a combination of the *energy-dense* and *bar* patterns considered in the present study. Rezazadeh, Rashidkhani and Omidvar [26] defined this combination as ‘not healthy’. Positive associations between patterns with compositions similar to those of the *energy-dense* and *bar* patterns and unfavorable health outcomes such as obesity have been described in some studies [9,14,30].

The differences in composition between patterns with the same denomination found in the literature, and the different denominations of factors of similar composition may be explained by the subjectivity inherent to the methods of principal component analysis [25]. In a review of this topic, Newby and Tucker [9] pointed out the following major decisions that should be made by an investigator which could influence the results: 1) which method to use for the investigation of food intake; 2) whether or not to group the food items; 3) how to treat the variables (grams/milliliters, % total caloric value, etc.); 4) number of retained factors; 5) starting from what value the factor loading will be considered to be important for the component; 6) how the retained patterns will be denominated. Newby and Tucker [9] also emphasize the importance of considering the nutritional composition of the foods that constitute each retained factor when attributing names to the dietary patterns identified. In the present study, the authors followed this recommendation.

Female sex and schooling in adulthood ≥12 years were found to be associated with the *healthy* dietary pattern. In contrast, men showed more adherence to the *traditional Brazilian* and *bar* patterns*,* as also observed in other studies that identified patterns of compositions similar to those studied here [16,26]. Differences in diet quality between sexes have been reported and it has been well established in the literature that women adhere more to healthy dietary patterns than men [9,11,17,25,26]. According to Newby and Tucker [9], this can be explained in part by the preoccupation of women with a good physical shape. A positive association between high schooling and income, and healthy food patterns has also been confirmed in other studies [11,17,26,27].

Mullatos showed greater adherence to the *traditional Brazilian* pattern, as it was also reported in other studies [3,16]. The fact that beans, the most important food in this pattern, were the principal diet component for those with low socioeconomic status during the colonial period of Brazil might explain this result. Also in agreement with other Brazilian studies [16,17], better socioeconomic levels at present (schooling and family income) and at birth (family income) showed an inverse association with this dietary pattern. The determinants of food choices are complex [34], and although price is not the only factor guiding these choices, a low income can limit them [11,34]. Except for beef, the foods composing the pattern called *traditional Brazilian* in the present study are of low cost. It should also be pointed out that only four food items presented an important positive factor loading in this pattern, suggesting monotony in the diet of less privileged socioeconomic classes, as also observed in a similar study [16].

Low schooling in adulthood was associated with the *bar* pattern, in contrast to other studies that identified patterns containing alcoholic beverages in their composition, where those of high schooling tended to show greater adherence to this pattern [16,30,35]. Low schooling may contribute to unhealthy food choices, since education allows people to obtain information about health, especially healthy dietary patterns, with consequent improvement of dietary habits [26].

Although none of the socioeconomic or demographic variables investigated showed association with the *energy-dense* pattern in the present study, female sex and higher schooling in adulthood and income in other population groups were associated with patterns of similar composition [16,17,30].

Social mobility was not associated with the healthy, bar and energy dense patterns. An interaction between sex and social mobility was detected only for the *traditional Brazilian* pattern. The category ‘not-poor - not poor’ showed lower adherence to the *traditional Brazilian* for both men and women. Similarly, young adults in the Brazilian city of Pelotas, of both sexes who were in the highest income tertile at birth and in adulthood (referred to as ‘never poor’), showed lower adherence to the ‘common Brazilian’ pattern compared to those who belonged to the lowest tertile of income at birth and remained there in adulthood, termed ‘always poor’. In the Pelotas study, the category ‘poor - not poor’ also showed greater adherence to the ‘processed food’ pattern compared to those who were ‘always poor’ [16]. Mishra et al. [31], studying British adults, found that those who held non-manual occupational activities and whose parents were also engaged in non-manual occupations at the time of their birth had healthier eating patterns than those who remained in the category manual as well as their parents.

In our study, the higher the number of people in the household the greater the adherence to the traditional Brazilian pattern. Also the adherence to this pattern was greater if the individual has had children. Demographic factors that take into account the number of household members have also been associated with some dietary patterns in other studies. For example, in Tehran, housing size > 20 m^2^/head was positively associated with a healthy dietary pattern [26].

The results of the present study should be compared with caution to those of other investigations since methodological differences exist between studies and food choices depend on socioeconomic and cultural factors of each population. Thus, the results of the present investigation cannot be generalized and only represent the dietary patterns of the cohort studied, but not of the entire population of young adults from Ribeirão Preto, São Paulo, Brazil. This is due to the fact that the cohort comprises a small age group born in a couple of years (1978/79). Furthermore, the population composition changed from birth to adulthood due to migration.

Our study has some limitations. It should be pointed out that there were selective losses when the group of individuals followed up in the fourth phase of the study was compared to subjects who were not followed up. The follow-up rates were slightly higher for women and for individuals whose mothers had higher schooling at the time of their birth. Although statistically significant, these differences were small [19]. Additional limitations are those inherent to the methods of dietary surveys and the subjectivity of the PCA method for the definition of dietary patterns [9]. Furthermore, PCA do not assume exactly an ordinal response option. In addition, the variability explained by the four retained components was relatively low (20.92%).

The FFQ used in this study was not validated in the present population, which is a limitation. However, it has been validated in second and third generation Japanese-Brazilian adults from São Paulo who have similar dietary habits to those of our population [22,36].

However, dietary surveys are widely used in epidemiological studies and, in the present investigation, the FFQ was applied by nutritionists with the assistance of a photo album in order to facilitate the estimation of portion sizes. In addition, all subjective choices typical of the PCA method were based on scientific knowledge and on an extensive review of the literature.

Thus, the present study allowed the identification of four major dietary patterns in this population of young adults and the identification of socioeconomic and demographic differences associated with food choices. In addition, it was possible to understand how socioeconomic factors existing at birth were associated with dietary patterns in young adulthood.

## Conclusions

Four principal dietary patterns were identified: *healthy, traditional Brazilian, bar* and *energy-dense*. Women and individuals with higher schooling showed greater adherence to the *healthy* pattern. The highest adherence to the *traditional Brazilian* pattern occurred for men, mullatos, households with ≥2 members and for those who have children, while individuals with higher schooling in adulthood, higher family income in adulthood, higher family income at birth and who were not poor at birth and remained so in adulthood showed lower adherence to this pattern. The *bar* pattern was positively associated with male sex and low schooling. Finally, the *energy-dense* pattern was not associated with any of the variables investigated.

## Abbreviations

MW: Minimum wages; FFQ: Food frequency questionnaire; KMO: Kaiser-Meyer-Olkin; PR: Prevalence ratios.

## Competing interests

The authors declare that they have no competing interests.

## Authors’ contributions

SPMA participated in the stages of data analysis and was responsible for writing the manuscript; AAMS participated in the stage of data analysis and critical review of the manuscript; GK and MG contributed to the discussion of the data and critical revision of the manuscript; HB and MAB were responsible for the original design of the project and participated in the discussion stages of the data and critical revision of the manuscript. All authors read and approved the final manuscript.

## Pre-publication history

The pre-publication history for this paper can be accessed here:

http://www.biomedcentral.com/1471-2458/14/654/prepub
